# Brain-controlled functional electrical stimulation therapy for gait rehabilitation after stroke: a safety study

**DOI:** 10.1186/s12984-015-0050-4

**Published:** 2015-07-11

**Authors:** Colin M. McCrimmon, Christine E. King, Po T. Wang, Steven C. Cramer, Zoran Nenadic, An H. Do

**Affiliations:** Department of Biomedical Engineering, University of California, Irvine, CA 92697 USA; Department of Neurology, University of California, Irvine, Orange, CA 92868 USA; Department of Anatomy & Neurology, University of California, Irvine, CA 92697 USA; Department of Physical Medicine & Rehabilitation, University of California, Irvine, Orange, CA 92868 USA; Department of Electrical Engineering and Computer Science, University of California, Irvine, CA 92697 USA

**Keywords:** Gait therapy, Stroke recovery, Brain computer interface, Electrical stimulation, EEG, Dorsiflexion

## Abstract

**Background:**

Many stroke survivors have significant long-term gait impairment, often involving foot drop. Current physiotherapies provide limited recovery. Orthoses substitute for ankle strength, but they provide no lasting therapeutic effect. Brain-computer interface (BCI)-controlled functional electrical stimulation (FES) is a novel rehabilitative approach that may generate permanent neurological improvements. This study explores the safety and feasibility of a foot-drop-targeted BCI-FES physiotherapy in chronic stroke survivors.

**Methods:**

Subjects (n = 9) operated an electroencephalogram-based BCI-FES system for foot dorsiflexion in 12 one-hour sessions over four weeks. Gait speed, dorsiflexion active range of motion (AROM), six-minute walk distance (6MWD), and Fugl-Meyer leg motor (FM-LM) scores were assessed before, during, and after therapy. The primary safety outcome measure was the proportion of subjects that deteriorated in gait speed by ≥0.16 m/s at one week or four weeks post-therapy. The secondary outcome measures were the proportion of subjects that experienced a clinically relevant decrease in dorsiflexion AROM (≥2.5°), 6MWD (≥20 %), and FM-LM score (≥10 %) at either post-therapy assessment.

**Results:**

No subjects (0/9) experienced a clinically significant deterioration in gait speed, dorsiflexion AROM, 6MWT distance, or FM-LM score at either post-therapy assessment. Five subjects demonstrated a detectable increase (≥0.06 m/s) in gait speed, three subjects demonstrated a detectable increase (≥2.5°) in dorsiflexion AROM, five subjects demonstrated a detectable increase (≥10 %) in 6MWD, and three subjects demonstrated a detectable increase (≥10 %) in FM-LM. Five of the six subjects that exhibited a detectable increase in either post-therapy gait speed or 6MWD also exhibited significant (*p* < 0.01 using a Mann–Whitney *U* test) increases in electroencephalogram event-related synchronization/desynchronization. Additionally, two subjects experienced a clinically important increase (≥0.16 m/s) in gait speed, and four subjects experienced a clinically important increase (≥20 %) in 6MWD. Linear mixed models of gait speed, dorsiflexion AROM, 6MWD, and FM-LM scores suggest that BCI-FES therapy is associated with an increase in lower motor performance at a statistically, yet not clinically, significant level.

**Conclusion:**

BCI-FES therapy is safe. If it is shown to improve post-stroke gait function in future studies, it could provide a new gait rehabilitation option for severely impaired patients. Formal clinical trials are warranted.

**Electronic supplementary material:**

The online version of this article (doi:10.1186/s12984-015-0050-4) contains supplementary material, which is available to authorized users.

## Introduction

Strokes are the leading cause of long-term disability in the U.S. with over 795,000 new cases each year [1], a number that will grow as the population ages and stroke survival rates increase. Despite physiotherapy and spontaneous recovery, ∼2 million stroke survivors in the U.S. suffer from long-term gait deficits. Post-stroke gait disability contributes to decreased participation in physical, social, and professional activities [2, 3], thereby exacerbating co-morbidities such as diabetes, cardiovascular disease, and depression [1].

The inability to dorsiflex the ankle during the swing phase of the gait cycle, known as foot drop, can contribute to gait problems, such as reduced walking speed [4, 5]. Many studies have shown that the use of an ankle-foot orthosis, which specifically corrects foot drop, improves gait velocity in stroke survivors [6–9]. Currently available orthoses and assistive gait devices (e.g. walkers and functional electrical stimulation [FES] devices) have not been shown to provide lasting effects after removal [10, 11]. Therefore, novel therapies that provide substantial, long-term gait improvement for stroke survivors are urgently needed.

There has been a growing interest in employing brain-computer interface (BCI) technology in post-stroke gait therapy [12–16]. BCIs use computers to translate signals of the central nervous system (e.g. via electroencephalography [EEG]) into control commands for external devices, providing peri-infarct areas with effector control in cortical strokes or bypassing the lesion in subcortical strokes. It has been hypothesized that by coupling the activation of upper motor neurons (UMNs) in the post-stroke cerebral cortex with the activation of α lower motor neurons (e.g. via functional electrical stimulation), lasting neurological and functional improvement may be achieved through a Hebbian learning process [17]. Utilizing a BCI ensures that patients are activating UMNs while receiving FES therapy, as opposed to passive (nonvolitional) electrical stimulation which may have less therapeutic potential [18]. Several reports on the implementation of brain-controlled FES systems [12–14] [19] and their application to stroke rehabilitation [13, 14, 20] have emphasized its promise as a new physiotherapeutic modality. However, no studies have systematically assessed safety and efficacy with behavioral outcomes, an important step before large-scale clinical trials can be performed. Although EEG and FES are quite safe separately, it is unknown whether they could promote maladaptive motor control and cause a deterioration in gait function when they are used in combination. For example, animal studies had shown that untimely coactivation of pre- and post-synaptic neurons can lead to long-term synaptic depression [21].

This Phase I clinical trial examined the safety of a novel foot-drop-targeted BCI-FES physiotherapy in a cohort of chronic stroke survivors using a previously developed system [12, 19]. Additionally, since this BCI-FES therapy may alleviate foot drop, and subsequently lead to an increased gait velocity [22], post-hoc analyses were performed to explore its potential efficacy and uncover any associated brain physiological changes.

## Methods

### Study design

**Fig. 1:**
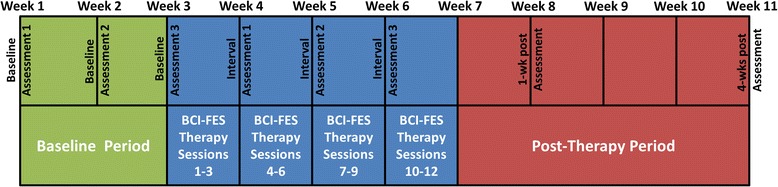
Schedule of activities for each subject. Interval Assessments 1, 2, and 3 were performed immediately before BCI-FES therapy sessions 4, 7, and 10, respectively

The study was approved by the University of California, Irvine, Institutional Review Board. Qualified chronic stroke subjects participated in 12 sessions of BCI-FES therapy for foot drop. Each subject first underwent the following baseline assessments: fast gait speed, dorsiflexion active range of motion (AROM), six-minute walk distance (6MWD), and Fugl-Meyer leg motor (FM-LM) score. The BCI-FES therapy was then administered at a rate of three one-hour sessions per week over the course of four weeks. The system was designed to detect when the subject was trying to dorsiflex his/her paretic foot, using EEG, and deliver electrical stimulation to the appropriate deep peroneal nerve. Neurological and functional assessments were performed immediately prior to every third session, as well as one week and four weeks after the 12^th^ session. The schedule of activities is summarized in Fig. 1. A before-and-after comparison was used to determine if any of the outcome measures deteriorated significantly.

### Subject recruitment

Advertisements were used to recruit stroke survivors (>6 mo post-stroke) with gait impairment that included foot drop. The entry criteria were: (1) age >18, (2) ability to walk ≥10 m without the use of an ankle-foot orthosis or assistance by another person (walking aids, e.g. a cane or walker, were permitted but had to be used consistently), (3) sufficient cognitive and language function to follow study-related instructions reliably. The exclusion criteria were: (1) absence of a tibialis anterior response to FES, (2) inability to tolerate FES, (3) presence of electronic implants, (4) severe spasticity or plantarflexion contracture (Modified Ashworth scale = 4). Although subjects were allowed to continue their personal exercise programs, initiating new rehabilitative treatments or physiotherapies during the study would result in exclusion from the study.

### BCI-FES dorsiflexion therapy

Qualified subjects underwent 12 one-hour long sessions of the experimental BCI-FES dorsiflexion therapy, generally performed at a rate of three sessions per week across four weeks. Before each session, the subjects were fitted with an EEG cap (10-10 International Standard), and impedances were reduced to <10 kΩ for a fixed 32-channel set. To provide the system with training EEG data, subjects followed 100 alternating six-second-long cues to relax or dorsiflex the paretic foot. Subjects were visually monitored for mirror movements (involuntary movements of a limb that are synchronous with the voluntary movements of the contralateral limb [23]) and co-contractions at other limbs, and were asked to discontinue these if they occurred. However, compliance with these instructions was not formally measured. The training data were recorded at 256 Hz with a NeXus-32 bioamplifier (Mind Media, Herten, Netherlands) to generate a session-specific decoding model for online operation (methods in [24–26]). This model could distinguish idling from dorsiflexion using EEG. Finally, surface FES electrodes were placed over the proximal course of the deep peroneal nerve (paretic side only), and stimulation parameters were adjusted to achieve ~15° dorsiflexion from the neutral position without discomfort.

During each one-hour-long session, subjects performed as many online BCI-FES runs as possible. Each run consisted of 10 alternating, contiguous 10-s-long dorsiflex/relax cues, during which the BCI-FES system detected the subjects’ intention to dorsiflex (or relax) from EEG and correspondingly provided (or withheld) stimulation. The accuracy of BCI operation was calculated as the percentage of correctly determined BCI-states at a rate of four decisions per second (decisions were calculated every 0.25 s based on the most recent 0.75 s of EEG data [24]).

Subjects were informed of the possibility of erroneous recognition of the idle and dorsiflexion states by the BCI. If FES was erroneously delivered when no movement was intended, subjects were instructed to ignore the stimulation and continue to relax; when movement was intended but no FES was delivered, subjects were instructed to continue to attempt dorsiflexion. Mirror movements and co-contractions were monitored visually. Brief breaks were provided between runs, or when requested, to prevent fatigue of the deep peroneal nerve and tibialis anterior muscle.

### Neurological and functional assessments

Neurological and functional measurements (fast gait speed, dorsiflexion AROM, 6MWD, and FM-LM score) were performed before, during, and after the BCI-FES therapy, and are defined below:

- **Gait Speed:** Fast gait speed [27] was measured [28] for the middle 6-m section of a 10-m walkway. This test was repeated 5 times at each assessment, and the average speed was calculated [29]. AFOs were removed, but walking aids such as walkers and canes were allowed. If a subject did use a walking aid at their first baseline assessment, they were asked to continue to use the same device throughout the rest of the study.
- **Dorsiflexion AROM:** The subject was placed in a seated position with the knee flexed at 90° and the tibial shank perpendicular to the ground. A goniometer was used to measure the AROM at the ankle as the subject dorsiflexed, using standard technique [30].
- **6 min Walk Distance:** Assessed as the distance that subjects can ambulate (at a safe, casual speed) in 6 min [31]. AFOs were removed, but walking aids were permitted.
- **Fugl-Meyer Leg Motor Score:** Assessed using the FM measurement system as defined in [32, 33].

Although chronic stroke subjects are assumed to have reached a plateau in terms of spontaneous behavioral recovery [34, 35], three baseline assessments (see Fig. 1) were performed to account for day-to-day variance [36]. Assessments were also performed immediately prior to every third BCI-FES session and again one week and four weeks after the 12^th^ session. Subjects were also asked to maintain a fall diary throughout the study. The results of this diary were documented at each weekly functional assessment.

### Outcome measures

All pre-stated outcome measures focused on safety. The primary outcome was the proportion of subjects who demonstrated a deterioration in gait speed ≥0.16 m/s at either the one-week or four-weeks post-therapy assessment. This threshold was chosen as it may be associated with a change in the modified Rankin Scale (mRS) for the post-stroke population [37]. The secondary outcome measures included the proportion of subjects who experienced a significant deterioration in dorsiflexion AROM, 6MWD, and FM-LM score at either the one-week or four-weeks post-therapy assessment. Deterioration of dorsiflexion AROM is defined as a ≥2.5° decrease from average baseline. There is no established minimum clinically important change in dorsiflexion AROM, so this threshold was chosen as it represents the minimal detectable difference [38]. The minimum clinically significant change in 6MWD is 20 % [39], while that of the FM-LM score is hypothesized to be 10 % [40].

### Post-Hoc analyses

Additional analyses were performed to determine the proportion of subjects who demonstrated a detectable increase in gait speed (≥0.06 m/s [37, 39]), dorsiflexion AROM (≥2.5°), 6MWD (≥10 % [41]), and FM-LM score (≥10 %) from average baseline at both post-therapy assessments. Detectable changes are not necessarily clinically important, so the proportion of subjects that experienced a clinically significant increase in gait speed (≥0.16 m/s) and 6MWD (≥20 %) was also calculated. Furthermore, the effect of BCI-FES therapy on gait speed, dorsiflexion AROM, 6MWD, and FM-LM was determined using independent linear mixed models (LMMs) with outcomes (gait speed, etc.) as a function of therapy group (pre-therapy baseline or post-therapy) with the random effects being subjects (and their interaction with therapy group) and repetitions within subjects (and their interaction with therapy group).

The training EEG datasets were analyzed for each session to determine if subjects experienced any brain changes throughout the course of the therapy. To reveal any spatial changes associated with therapy, the dorsiflexion-related importance (the μ-measure defined in [42]) of each EEG channel was plotted for all training sessions (details in Additional file 1). Additionally, changes in event-related synchronization (ERS) and event-related desynchronization (ERD) throughout the therapy were analyzed as follows. The most important channel (highest average μ across all sessions) was identified for each subject, and the EEG data from this channel were aggregated by week. The median ERS and ERD at each frequency (8–30 Hz in 2 Hz bins) were calculated for weeks two through four (Equations 2 and 3 in Additional file 1) and compared to their respective week-one value using repeated Mann–Whitney U tests (Bonferroni corrected α = 0.01).

## Results

### Overview

Nine subjects provided their informed consent to participate in the study (Table 1). All subjects completed the baseline and interval assessments, 12 sessions of BCI-FES therapy, and the one-week post-therapy assessment. Subject S1 suffered a recurrent stroke after the one-week post-therapy assessment, and therefore the four-weeks post-therapy assessment could not be obtained. Subject S2 could not be contacted for the four-weeks post-therapy assessment. Subject S6 experienced leg pain during Interval Assessment 2 and declined the 6MWD test. The subjects averaged 8.4 BCI-FES runs per session. Almost 95 % of the total number of BCI-FES runs across all subjects and sessions were at a significant performance level (α = 0.01) compared to Monte-Carlo simulation (details in [24]; see Table 1).

**Table 1:** Demographic and BCI performance data for all subjects

Subject	Age/Sex	Time Since Stroke (mo)	Stroke Type	Stroke Location	Clinical Presentation	NIH Stroke Scale	Barthel Index	Geriatric Depress ion Score	FM-LM Score (first/last assessm ent)	Walking Aids Used at Time of Study	Previous Lower Limb FES Use	BCI-NMES Runs Completed^a^	Overall Decoding Accuracy (%)
S1	83/M	29	I	L basal ganglia	R hemiparesis	5	90	0	23/27	AFO	N	107	82.7
S2	59/F	9	I	R internal capsule	L hemiplegia	8	60	4	21/25	AFO + C	N	98	79.1
S3	35/M	24	H	R corona radiata	L hemiparesis	6	100	0	29/31	AFO	Y	119	61.6
S4	75/M	9	I	L putamen/corona radiata	R hemiparesis	7	75	7	18/21	AFO + W	N	111	74.5
S5	51/M	8	I	L basal ganglia	R foot drop	3	100	6	25/26	AFO	N	113	84.2
S6	71/M	102	I	R basal ganglia	L hemiparesis	2	100	1	25/29	AFO	N	97	75.5
S7	66/F	23	I	L pons	R hemiparesis	5	95	4	21/26	AFO + C	N	103	86.9
S8	38/F	24	H	R basal ganglia	L hemiparesis	5	70	1	23/22	AFO + C	N	80	84.1
S9	60/M	19	H	R thalamus	L hemiparesis	5	100	2	26/26	AFO	N	82	88.6

*M:* male, *F:* female, *I:* ischemic, *H:* hemorrhagic, *L:* left, *R:* right *AFO:* ankle-foot orthosis, *C:* cane, *W:* walker, *Y:* yes, *N:* no

^a^Out of a total of 910 runs, 864 were deemed significant (α = 0.01) by comparison to Monte-Carlo Simulation

### Primary outcome measure

**Fig. 2:**
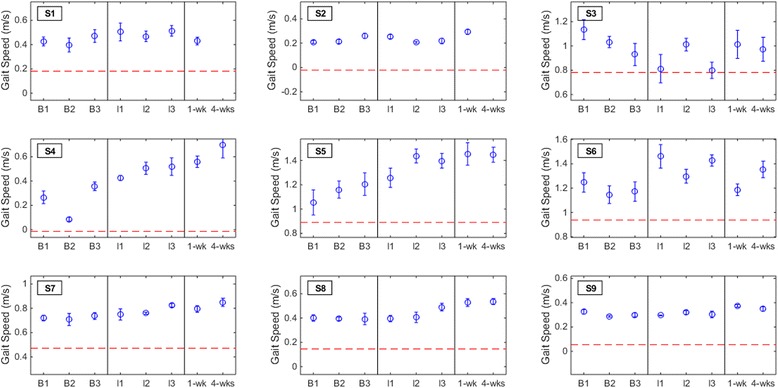
Gait speed measurements for each subject. The results from the baseline (B1, B2, and B3), interval (I1, I2, and I3), and post-therapy (one week and four weeks) gait speed assessments are shown for all subjects. The error bars shown here represent the within-assessment standard deviation (gait speed measurements were repeated five times per assessment), while the blue circles represent the within-assessment means. Note that the error bars are only displayed here to provide insight into the within-assessment standard deviation, and only the means for each assessment [29] were used in the outcome measures and post-hoc analyses. The red dashed line denotes the threshold of clinically important deterioration (0.16 m/s decrease from average baseline)

No subjects demonstrated a clinically significant decrement in gait speed at either the one-week (subjects S1-S9) or the four-weeks (subjects S3-S9) post-therapy assessment (see Fig. 2). The test-retest reliability [43, 44] of the gait speed measurements, >0.98, was comparable to reported values [41, 45]. Post-hoc analysis revealed that five out of nine subjects (56 %) exhibited a detectable increase in gait speed at both post-therapy assessments. S9 experienced a detectable increase in gait speed at one week post-therapy that disappeared by the last assessment. S6 experienced a detectable increase in gait speed only at four weeks post-therapy. Additionally, two of nine subjects (22 %) experienced a clinically important increase in gait speed (≥0.16 m/s) at both post-therapy assessments. S6 experienced a clinically important increase in gait speed only at four weeks post-therapy. Note that although these increases may be clinically relevant, they are not necessarily statistically significant. With a pre-post therapy slope of 0.025 (*p* ≤ 0.03; intercept = 0.63), the LMM demonstrated that BCI-FES therapy may be associated with an increase in gait speed.

### Secondary outcome measures

**Fig. 3:**
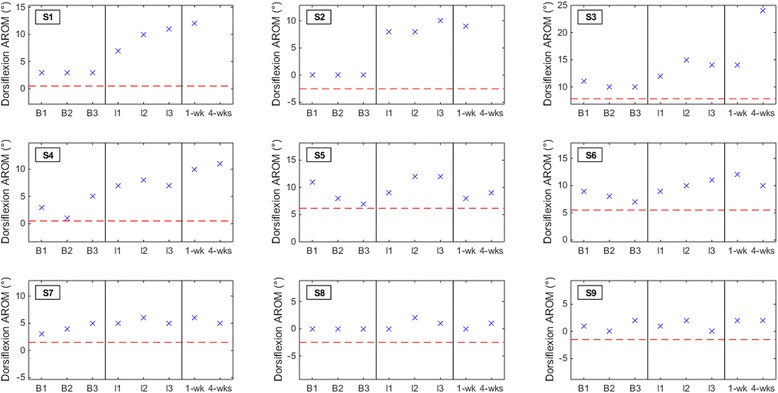
Dorsiflexion active range of motion (AROM) results for all subjects at each baseline (B1, B2, and B3), interval (I1, I2, and I3), and post-therapy (one week and four weeks) assessment. The red dashed line denotes the threshold of clinically detectable deterioration (2.5° decrease from average baseline)

**Fig. 4:**
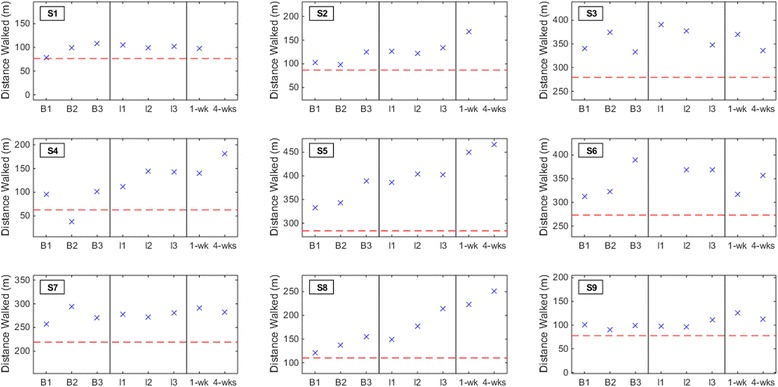
Subjects’ six minute walking distance (6MWD) at each baseline (B1, B2, and B3), interval (I1, I2, and I3), and post-therapy (one week and four weeks) assessment. The red dashed line denotes the threshold of clinically important deterioration (20 % decrease from average baseline)

**Fig. 5:**
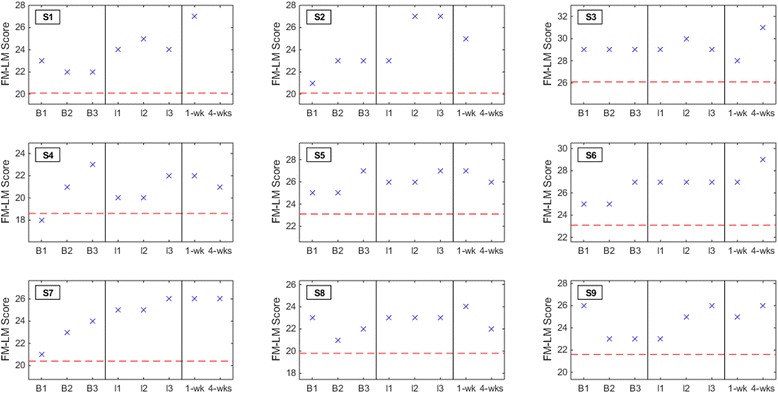
Subjects’ Fugl-Meyer leg motor (FM-LM) scores at each baseline (B1, B2, and B3), interval (I1, I2, and I3), and post-therapy (one week and four weeks) assessment. The red dashed line denotes the threshold of clinically detectable/important deterioration (10 % decrease from average baseline)

No subjects experienced a deterioration in dorsiflexion AROM, 6MWD, or FM-LM score at either the one-week or four-weeks post-therapy assessment (Figs. 3, 4 and 5). Post-hoc analysis revealed that three out of nine subjects (33 %) had a detectable (≥2.5°) increase in dorsiflexion AROM at both post-therapy assessments. Five subjects (out of nine) had detectable increase by the first post-therapy assessment, while only two subjects (out of seven) retained this increase at four weeks post-therapy. With a pre-post therapy slope of 0.84 (*p* = 0.013; intercept = 4.22), the LMM demonstrated that BCI-FES therapy may be associated with an increase in dorsiflexion AROM. The 6MWD increased by ≥10 % from average baseline for five subjects (56 %) and ≥20 % for four subjects (44 %) at the post-therapy assessments. S9 experienced a detectable increase in 6MWD at both post-therapy assessments, but only the increase at one week post-therapy was also clinically important. With a pre-post therapy slope of 8.52 (*p* = 0.02; intercept = 204.10), the LMM demonstrated that BCI-FES therapy may be associated with an increase in 6MWD. FM-LM score increased by ≥10 % for subjects S1, S2, and S7 (33 % of subjects) at the post-therapy assessments. S6 only experienced an increase in FM-LM at the four-weeks post-therapy assessment. Once again, changes in dorsiflexion AROM, 6MWD, and FM-LM scores per subject may be clinically significant, but they are not necessarily statistically significant. With a pre-post therapy slope of 0.36 (*p* < 0.001; intercept = 23.81), the LMM demonstrated that BCI-FES therapy may be associated with an increase in FM-LM score.

### Adverse events

Subject S1 reported a recurrent stroke after his one-week post-therapy assessment. The cause of stroke was idiopathic, but was suspected to be associated with a prosthetic heart valve, and was thereby deemed unrelated to the BCI-FES therapy. Subject S7 reported a fall (not resulting in serious injury) while carrying a heavy object after the one-week post-therapy assessment. This was also considered unrelated to the study procedures. No other adverse events, such as peroneal nerve dysesthesias or skin breakdown, were reported.

### EEG changes during therapy

**Fig. 6:**
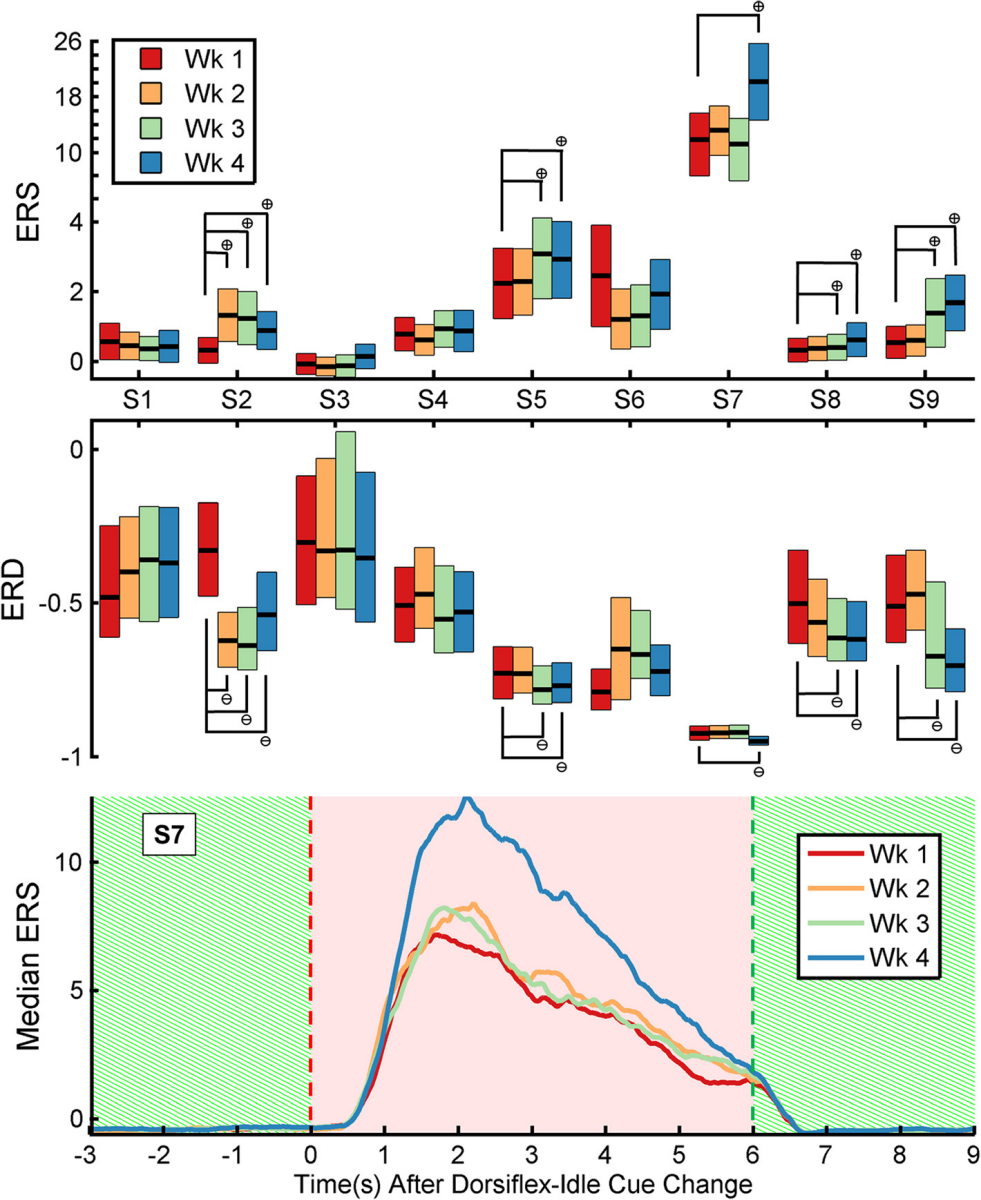
Top and Middle: Event-related synchronization (ERS) and desynchronization (ERD) during each week for all subjects. ERS, top, and ERD, middle, during weeks one through four (wk 1–4, or sessions 1–3, 4–6, 7–9, 10–12, respectively). Boxes signify intervals of one median absolute deviation around the median (central black bar). Significant positive and negative changes from week one are denoted by a cross-in-circle symbol and a line-in-circle symbol, respectively. Bottom: Temporal profile of median ERS during weeks one through four, taken from S7. The cue being presented is denoted by the background (“Dorsiflex”, hatched green; “Relax”, solid red)

Five out of the six subjects that exhibited a detectable improvement in post-therapy gait (increase in gait speed or 6MWD) also exhibited a significant increase in ERS and ERD (83.3 % sensitivity). The remaining three subjects demonstrated no significant increase in ERS or ERD (100 % specificity). The evolution of ERS and ERD are shown in Fig. 6.

**Fig. 7:**
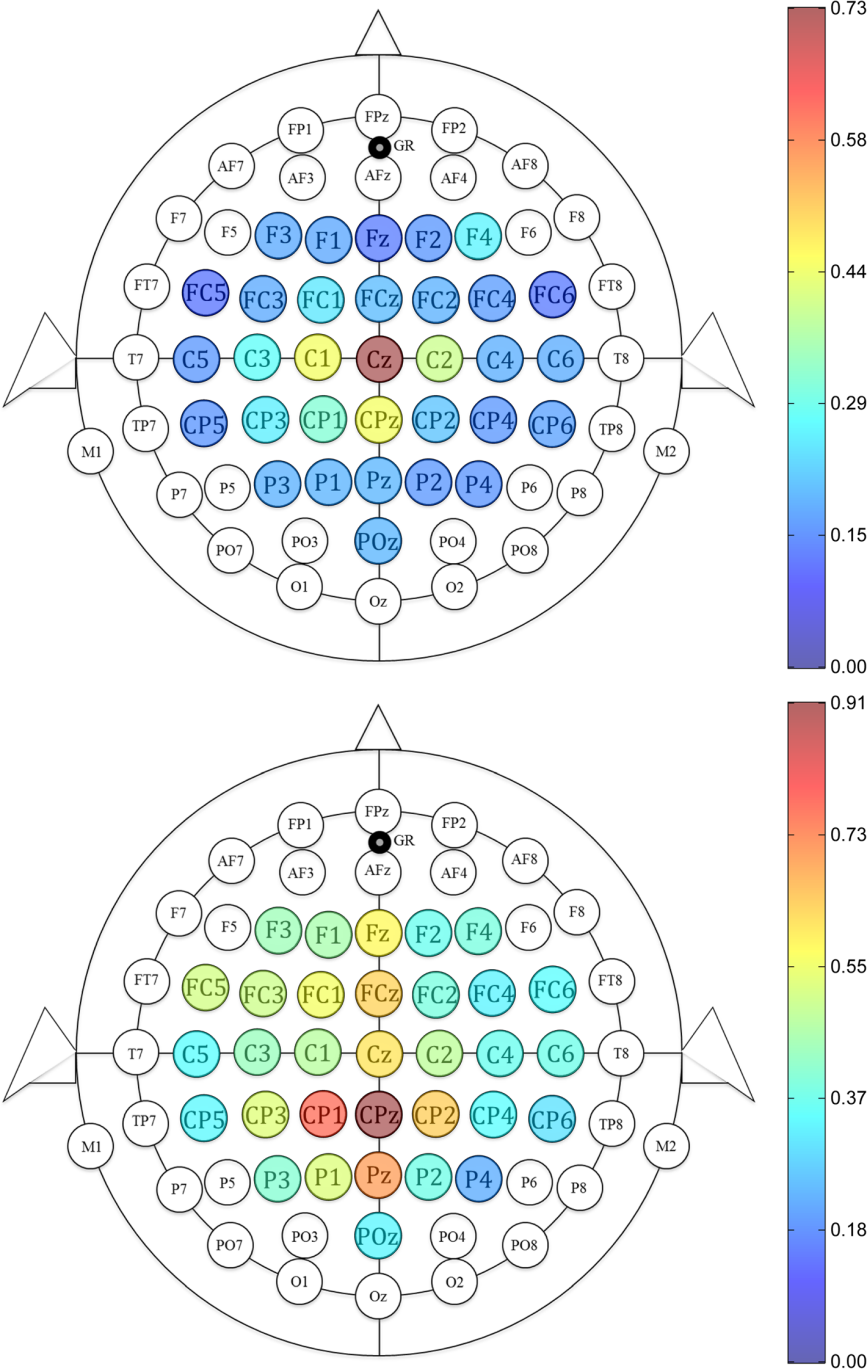
Importance of each EEG channel for dorsiflexion. Channels are colored based on their μ value, from dark blue (unimportant) to dark red (highly important). These two representative maps are taken from subject S8 (top) and S9 (bottom)

For subjects S1, S2, S4, S5, S6, S7, and S8, the most salient EEG channel for distinguishing idling from dorsiflexion (highest μ value) was Cz; for S3 and S9, it was C5 and CPz, respectively. Representative maps of the EEG channels’ importance for dorsiflexion are shown in Fig. 7 (additional plots in Additional file 1). No subject demonstrated a consistent change in the location of his/her most salient EEG channel throughout the BCI-FES therapy.

### Subjective reports

Subject S2 indicated at the one-week post-therapy assessment that she could climb stairs for the first time since her stroke. Subject S4 indicated that his newfound gait improvement allowed him to walk farther during his daily exercises. Subject S5 stated that he had better control of the gas pedal while driving after beginning the BCI-FES therapy. Although subject S6 felt that the therapy was somewhat tedious, he was enthusiastic about his improvement in dorsiflexion AROM. Subject S7 felt that she had more consistent control of foot dorsiflexion and knee flexion/extension. Subject S9 reported an increased sensation and strength in his paretic side, leading to what he considered to be a more natural gait. All reports were provided or solicited informally at the end of the study.

## Discussion

No clinically important deterioration in gait speed, dorsiflexion AROM, 6MWT distance, or FM-LM score was present one week or four weeks after completing the BCI-FES therapy. Throughout the study, only one fall was reported, an incidence well below that of conventional outpatient stroke rehabilitation [46–48]. The lack of deterioration in the measured gait characteristics indicates that the BCI-FES dorsiflexion therapy may be safe to explore in larger populations of stroke survivors. In addition, post-hoc analysis revealed that five subjects were walking detectably faster (≥0.06 m/s) at the post-therapy assessments. Two of these subjects were walking ≥0.16 m/s faster than average baseline, quite a remarkable feat since this magnitude is associated with a ≥1 increase in mRS [37]. Furthermore, the data suggest that BCI-FES therapy is associated with a statistically significant, albeit not clinically significant, increase in gait speed, dorsiflexion AROM, 6MWD, and FM-LM score. Since this study focused on safety outcomes and was not designed to test efficacy, any improvement in gait function cannot yet be attributed to the BCI-FES therapy. Nonetheless, given that increased gait speed is strongly associated with increased social re-integration after stroke [2], and based on this therapy’s acceptable early safety profile, larger clinical trials are warranted to definitively establish its safety and efficacy.

Given that the BCI-NMES system was inspired by the concept of Hebbian learning between M1 and foot dorsiflexion motor pools [17], increased dorsiflexion AROM and associated brain changes were expected. However, increased dorsiflexion AROM generally did not persist at four weeks post-therapy (verified via an insignificant LMM slope using post-therapy data from only this assessment), and ERS and ERD changes occurred, instead, in association with increased gait speed alone. These increases in ERS and ERD indicate that activation of foot and leg sensorimotor areas is more synchronous (Fig. 7 and Additional file 1), and suggest the presence of an underlying neural process. Possible mechanisms include Hebbian learning between UMNs and the spinal cord gait central pattern generators [49] and increased afferent sensory feedback during electrical stimulation and walking [50]. It also remains a possibility that modest increases in dorsiflexion strength (via the Hebbian learning mechanism expected by the authors) may have occurred, but subsequent increases in dorsiflexion AROM were obscured by mild plantar flexion contractures. Ultimately, formal physiological studies are needed to further elucidate the underlying mechanism.

Of note, the authors believe that subject S6 experienced problems with walking during the one-week post-therapy assessment due to a new pair of poorly fitting shoes. This is supported by the presence of a transient drop in his gait speed and 6MWD. By four weeks post-therapy, these measures surpassed their baseline values.

Lastly, at the start of the study the mean baseline gait speed and 6MWD were 0.63 ± 0.40 m/s and 204.1 ± 122.5 m, respectively, lower than that of the chronic stroke population reported in [51]. One potential strength of BCI-based movement therapies is that they are accessible to individuals with baseline motor functions too low for other interventions (e.g. treadmill training).

### Limitations

The major limitation of this study is the small sample size and the lack of matched controls and unbiased raters. Additionally, large, controlled studies, i.e. Phase III clinical trials, are necessary to definitively establish efficacy. However, the small sample size is appropriate for an initial investigation into the safety of this BCI-FES therapy. Furthermore, all subjects were assumed to have reached a steady rehabilitative state (>6 months post-stroke) [34], and were thus used as their own controls. Also, baseline assessments for all subjects appeared steady. Since the majority of subjects had subcortical strokes, particularly in the basal ganglia, it will be important to examine how BCI-FES therapy affects those with cortical strokes in future studies. Ankle dorsiflexion was chosen as the target of FES therapy since studies have concluded that foot drop plays a role in post-stroke gait impairment [4, 5]. On the other hand, studies such as [52] stress the important role of other muscle groups in post-stroke gait impairment. However, the BCI-FES therapy implemented in this study can easily be applied to other muscle groups. Finally, although multiple meta-analyses [10, 11] suggest that functional electrical stimulation alone does not provide a conclusive long-term therapeutic effect, a formal direct comparison with BCI-FES therapy may be necessary in the future.

## Conclusions

Since this BCI-FES therapy appears to be safe and a large proportion of subjects experienced improvements in gait speed and dorsiflexion AROM, larger controlled studies (including pre-clinical and Phase II/III studies) are warranted to investigate additional aspects of this novel therapy. These include: (1) the potential efficacy and optimal duration of BCI-FES therapy, (2) the neurobiological principles that underlie any functional changes, (3) the subpopulation of stroke patients that will benefit most, and (4) any synergism with conventional physiotherapies.

## Additional file

Additional file 1: **Supplementary material and figures.**
