# Supplementary material for: Brain-controlled functional electrical stimulation therapy for gait rehabilitation after stroke: a safety study
Source: J Neuroeng Rehabil. 2015 Jul 11;12:57. doi: 10.1186/s12984-015-0050-4 (PMC4498504; doi:10.1186/s12984-015-0050-4)
Supplement: Supplementary file 1 — Supplementary material and figures. [file 12984_2015_50_MOESM1_ESM.pdf]

SUPPLEMENTARY MATERIAL
Brain-controlled functional electrical stimulation therapy for gait rehabilitation after stroke: a safety study

May 22, 2015

Colin M. McCrimmon[1], Christine E. King[1], Po T. Wang[1], Steven C. Cramer[2,3,4], Zoran Nenadic[1,5], An H. Do[2]

1. Department of Biomedical Engineering, University of California, Irvine, Irvine, CA 92697
2. Department of Neurology, University of California, Irvine, Orange, CA 92868
3. Department of Anatomy & Neurology, University of California, Irvine, Irvine, CA 92697
4. Department of Physical Medicine & Rehabilitation, University of California, Irvine, Orange, CA 92868
5. Department of Electrical Engineering and Computer Science, University of California, Irvine, Irvine, CA 92697

1 Supplemental Methods

1.1 Dorsiflexion-Related Importance or the μ-Metric

The μ-metric [1] was calculated for each channel (c) and frequency bin (f) as:

$$\mu_{c,f} = \frac{1}{4} \ln \left(\frac{\sigma^2}{\sigma_{Relax}\sigma_{Dorsiflex}} \right) \tag{1}$$

where σ_{Relax} and $\sigma_{Dorsiflex}$ are the variances in EEG power during relaxation and dorsiflexion of the ankle respectively, and σ is the total variance in EEG power.

1.2 Spatial Maps of Dorsiflexion-Related Importance

The frequencies important to dorsiflexion for each subject may not be broadly distributed. Thus, to determine if subjects' most salient EEG channel for foot dorsiflexion changed throughout the study, the maximum μ value across all frequency bins was calculated from the training data of each BCI-FES session. This ensured that these salient features were not lost. The resulting values from each session were either (1) normalized to the highest (per session) value and displayed in Figure I or (2) averaged across sessions to create average dorsiflexion-related importance plots (Figures 7 [main text] and II).

1.3 ERS and ERD Calculation

At each channel and frequency bin, the ERS and ERD of the kth epoch were calculated as follows:

$$ERS_k = \frac{P_k - \mathbb{E}[P_{Dorsiflex}]}{\mathbb{E}[P_{Dorsiflex}]} \tag{2}$$

$$ERD_k = \frac{P_k - \mathbb{E}[P_{Relax}]}{\mathbb{E}[P_{Relax}]} \tag{3}$$

where P_k is the average power of the kth epoch, and the expected values ($\mathbb{E}[P_{Dorsiflex}]$ and $\mathbb{E}[P_{Relax}]$) represent the mean power during all "Dorsiflex" and "Relax" epochs respectively. Note that the time-resolved (not time-averaged) power of the kth epoch, $P_k(t)$, was used to calculate the time resolved ERS plot in Figure 4 (main text).

2 Supplemental Figures

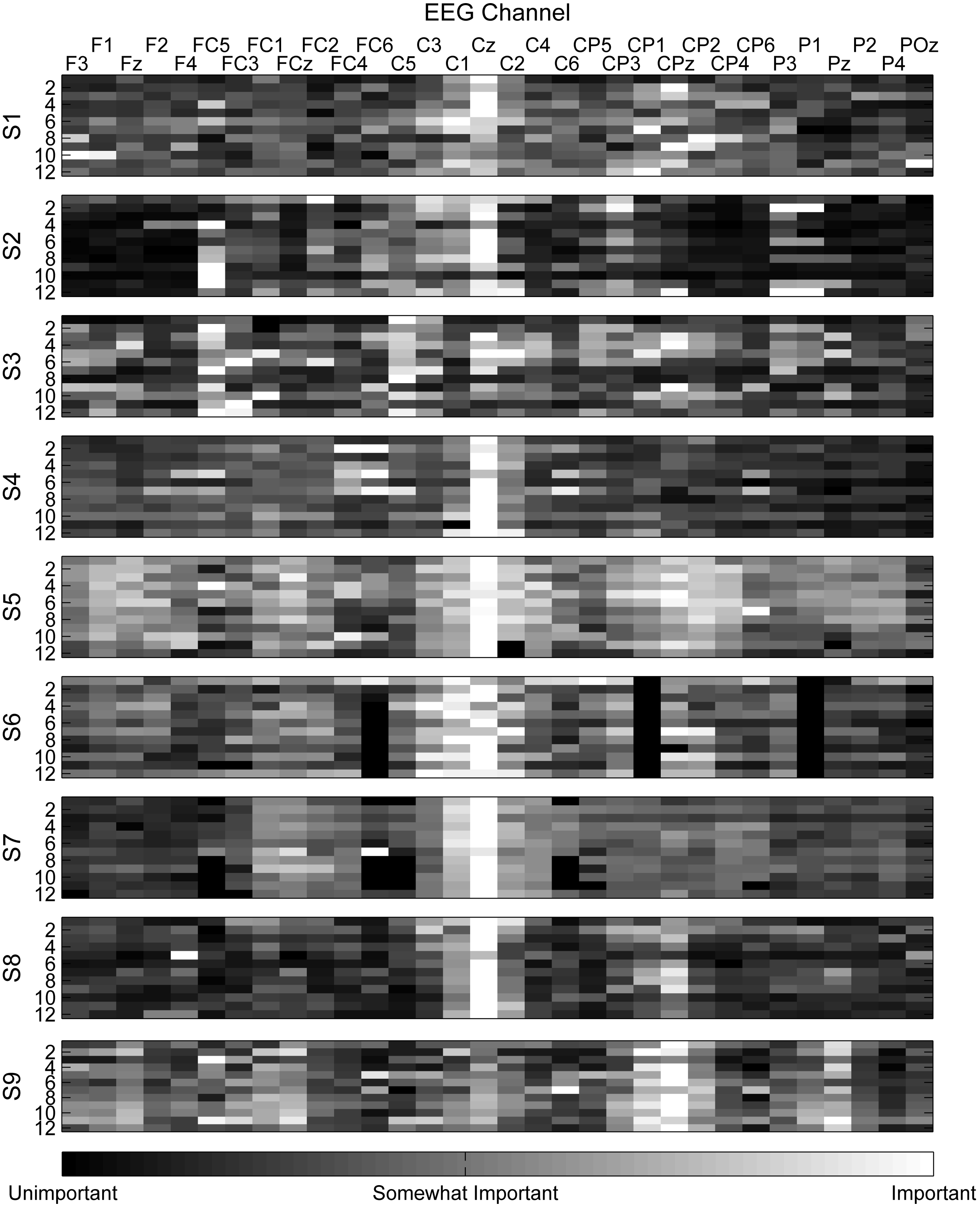

Figure I: Relative importance of each EEG channel for all training sessions. Highly important channels appear white, while channels that are unrelated to foot dorsiflexion appear black. Note that channel Cz is highly important to most subjects' dorsiflexion, and that no subjects exhibited consistent changes in their "important" channels.

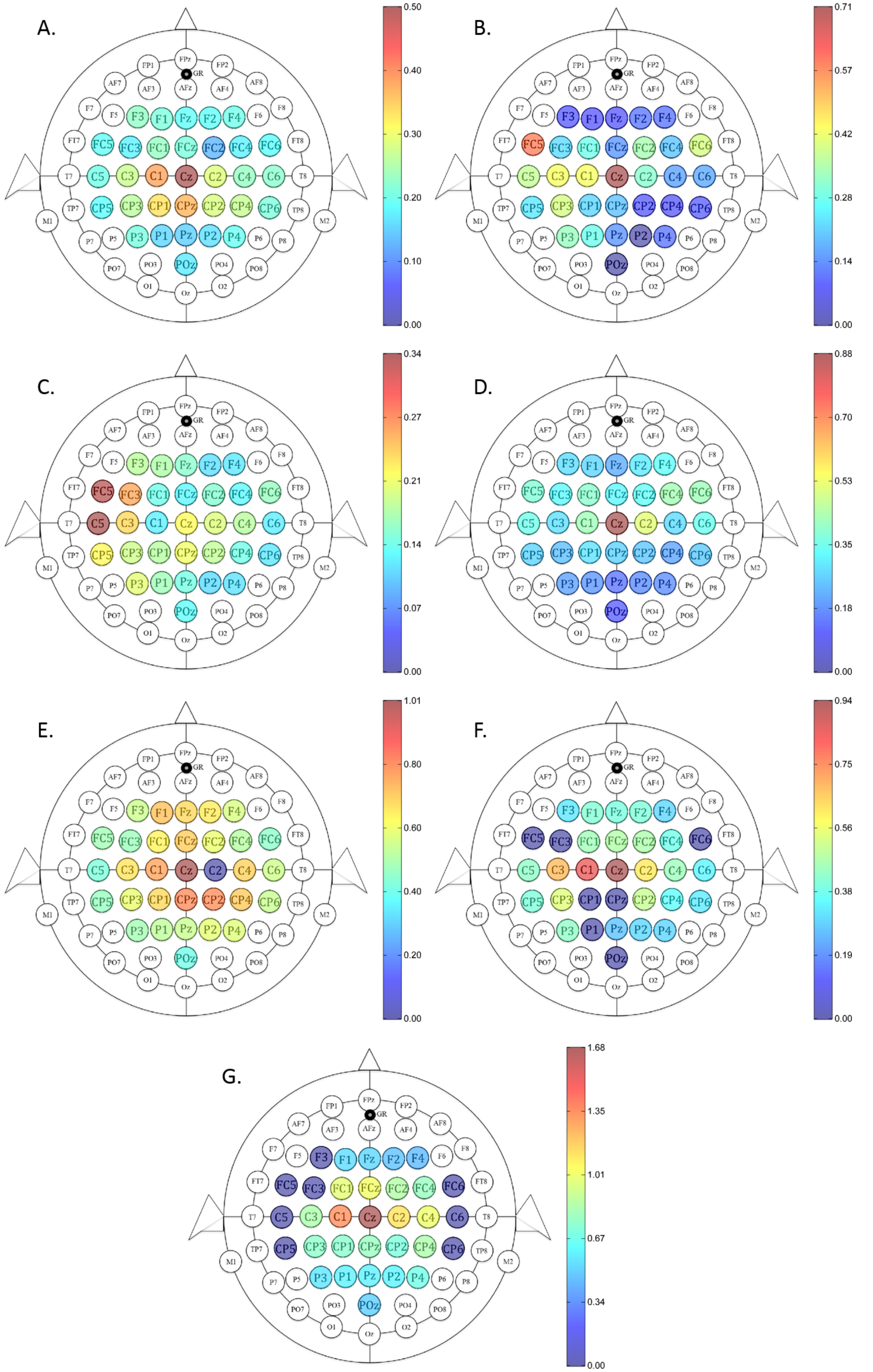

Figure II: Map of the importance of each EEG channel for subject S1's (A), S2's (B), S3's (C), S4's (D), S5's (E), S6's (F), and S7's (G) control of dorsiflexion, from unimportant (dark blue) to highly important (dark red).

Supplemental References

[1] Nenadic Z. Information Discriminant Analysis: Feature Extraction with an Information-Theoretic Objective. 2007;29(8):1394–1407.
